# New Insights into Placozoan Sexual Reproduction and Development

**DOI:** 10.1371/journal.pone.0019639

**Published:** 2011-05-19

**Authors:** Michael Eitel, Loretta Guidi, Heike Hadrys, Maria Balsamo, Bernd Schierwater

**Affiliations:** 1 Stiftung Tierärztliche Hochschule Hannover, ITZ, Ecology and Evolution, Hannover, Germany; 2 Department of Earth, Life and Environment Sciences, Scientific Campus, Urbino, Italy; 3 Department of Molecular, Cellular and Developmental Biology, Yale University, New Haven, Connecticut, United States of America; Laboratoire Arago, France

## Abstract

Unraveling animal life cycles and embryonic development is basic to understanding animal biology and often sheds light on phylogenetic relationships. A key group for understanding the evolution of the Metazoa is the early branching phylum Placozoa, which has attracted rapidly increasing attention. Despite over a hundred years of placozoan research the life cycle of this enigmatic phylum remains unknown. Placozoa are a unique model system for which the nuclear genome was published before the basic biology (i.e. life cycle and development) has been unraveled. Four organismal studies have reported the development of oocytes and one genetic study has nourished the hypothesis of sexual reproduction in natural populations at least in the past. Here we report new observations on sexual reproduction and embryonic development in the Placozoa and support the hypothesis of current sexual reproduction. The regular observation of oocytes and expressed sperm markers provide support that placozoans reproduce sexually in the field. Using whole genome and EST sequences and additional cDNA cloning we identified five conserved sperm markers, characteristic for different stages in spermatogenesis. We also report details on the embryonic development up to a 128-cell stage and new ultrastructural features occurring during early development. These results suggest that sperm and oocyte generation and maturation occur in different placozoans and that clonal lineages reproduce bisexually in addition to the standard mode of vegetative reproduction. The sum of observations is best congruent with the hypothesis of a simple life cycle with an alternation of reproductive modes between bisexual and vegetative reproduction.

## Introduction

The Placozoa have formerly and recently attracted much attention in the context of characterizing the ancestor of all metazoans, the Urmetazoon. According to Bütschli's placula hypothesis metazoan life started with a single two-layered benthic organism, the placula, which reproduced both vegetatively and sexually. While from a morphological point of view the scenario *cum grano salis* looks straight forward, molecular systematics has not resolved the phylogeny at the base of the metazoan tree of life yet. Different phylogenetic state different scenarios for the early evolution of the Metazoa [1]–[5].

Fundamental for Bütschli's “placula hypthesis” of metazoan evolution was the morphological simplicity of *Trichoplax adhaerens*, the only nominal species within the phylum Placozoa [6]–[10]. *Trichoplax* has only five somatic cell types, lacks any kind of symmetry and has no extra cellular matrix and no nerve or muscle cells [6], [11], [12]. Thus *Trichoplax* is the simplest organized animal from a morphological perspective [12], [13]. The phylum Placozoa has been entering a pivotal position in many areas of modern biology [14]. It is the only phylum for which a complete nuclear genome was published [15] without knowledge of the life cycle and basic biology. Studying sexual reproduction and embryonic development in the Placozoa will be quite crucial not only for identifying the Urmetazoon but also for using the Placozoa as a model system for future studies in all areas of biology.

The question whether placozoans reproduce sexually in the field has not been answered yet. One study has provided molecular evidence for sexual events by uncovering allele shuffling, thus indicating a sexual life cycle at least in the past [16]. Sexually reproducing animals have not yet been identified in the field. Nonetheless, embryonic development has been studied to some extent in the laboratory [17]–[21]. Under laboratory conditions, *Trichoplax adhaerens* usually propagates clonally by binary fission and sometimes by producing buds, the so-called swarmers [22]–[24]. Kept at high animal densities and with food scarceness, however, female gametes (oocytes) are built within 4–6 weeks [18], [20]. These only appear in so-called D-phase ( = degeneration phase) animals and are always accompanied by the accumulation of big droplets of ‘fatty substances’ [18], [20]. The oocytes are possibly derivates of the lower epithelium [20]. Through incorporation of extensions from nursing fiber cells attached to their surface, they grow into the inter spaces between the lower and upper epithelium. After reaching a varying mature size of 70–120 µm oocytes are fertilized. Following fertilization the so-called ‘fertilization membrane’ (FM), a protective eggshell, is built around the zygote which starts total, equal cleavage [18]. Early embryos grow inside the mother animal until the latter completely degenerates and releases the embryo. Male gametocytes (sperm) were also described in an ultrastructural study [12] but their functionality was not confirmed.

Although substantial efforts have been made to follow embryonic development, embryos never developed beyond a 64-cell stage [20], [21]. As a reason for the cease in embryonic development an uncontrolled DNA replication was claimed, preventing the switch from S-phase to the G_2_-phase of the cell cycle [21] and pruning the embryo to die. Throughout the embryonic development no nuclei were found as the nucleus undergoes fragmentation before the fertilization membrane is formed [21]. The authors claimed that this observation may be an artifact of laboratory conditions and that degeneration must not necessarily take place in naturally reproducing animals.

Here we provide molecular hints for the existence of spermatogenesis and sperm maturation in placozoans. In addition we describe in-depth analyses of growing oocytes and embryos from a placozoan representative by means of fluorescence, confocal laser scanning and electron microscopy. We also report further culturing improvements leading to the identification of nuclei and chromosomes in the embryos under laboratory conditions allowing embryos to develop at least to a 128-cell stage. While all former studies on Placozoa were performed on *Trichoplax adhaerens*, still the only valid species in the phylum, we here report data from different species-lineages.

## Results

### Induction of sexual reproduction

**Figure 1:**
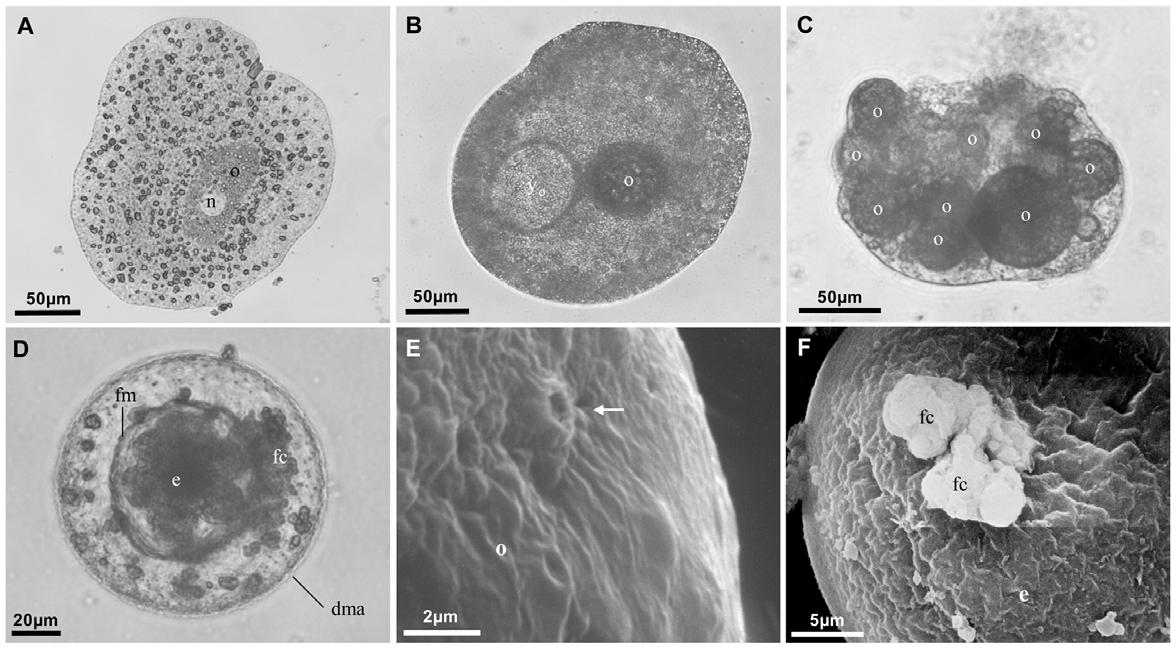
Progress of Placozoa sp. H2 oocyte maturation and early embryogenesis. Shown are light microscopy (A–D) and SEM (E, F) images of Placozoa sp. H2 oocytes and embryos. Typically, one oocyte with a large nucleus starts growing in a flat animal without any signs of degeneration (A). Accompanied by the generation of yolk droplets (B) the animal enters the degeneration phase (D-phase) after 5–6 weeks when the population density reaches its maximum. Occasionally several oocytes are found in a single degenerating animal. We found one animal with nine maturing oocytes (C). The D-phase starts with the lifting of the upper epithelium followed by condensing the lower epithelium until forming a hollow sphere (“brood chamber”) containing the embryo (D, compare Fig. 3a1). The oocyte grows until reaching a varying final size of 50–120 µm by incorporating extensions from fiber cells through pores. One ‘connection pore’ of a maturing oocyte is shown in (E) (arrow). After fertilization the protective ‘fertilization membrane’ (eggshell) is built around the zygote (D), which starts total equal cleavage. Often formerly nursing fiber cells are still attached to the fertilization membrane (D, F). n = nucleus, o = oocyte, y_o_ = yolk outside oocyte, fm = fertilization membrane, e = embryo, fc = fiber cells, dma = degenerating mother animal.

We have induced sexual reproduction in three placozoan lineages (putative species; cf. [25]): *Trichoplax adhaerens*, Placozoa sp. H2 and Placozoa sp. H16 (see [25]–[27]) for details on phylogenetic relationships in Placozoa). The criterion for successful induction of sexual reproduction was the maturation of oocytes. We tested different food sources, salt concentrations and temperatures to optimize conditions necessary for triggering sexual reproduction. The only factor that consistently affected the induction of oocyte maturation was the temperature with oocytes being built only at 23°C or more. In Placozoa sp. H2, which was studied in detail, oocyte maturation and early embryonic development resemble that of *Trichoplax adhaerens* ([18], [20]; see Figure 1): high population density and food depletion led to the development of oocytes and to the accumulation of yolk outside the oocyte. Growth of maturing oocytes was always accompanied by the degeneration of the mother animal. The degeneration process starts by the animal lifting the upper epithelium and condensing the lower epithelium. This leads to a round-shaped animal harboring the oocyte and later on the embryo in the center. Because of the circular shape, the animal loses its contact to the surface and slides over the bottom by ciliary movements. More than 70% of all female animals developed a single oocyte that always gave rise to an embryo. Animals with more than one oocyte usually absorbed the extra oocytes. In some rare cases we observed up to three embryos developing in a single mother animal.

### Identification of sperm-specific markers

To identify sperm-associated genes in the Placozoa we used EST and whole genome data from *Trichoplax adhaerens*, EST data from Placozoa sp. H4 and target gene amplifications from a Placozoa sp. H2 cDNA library. For target gene cloning healthy growing adult animals with no sign of degeneration were used to prepare cDNA libraries (see [28]). Five candidate sperm-associated proteins were identified using our Blast-based reciprocal search method: Spag8, Dnajb13, Mns1, Meig1 and Nme5 (Table 1). All five proteins are present in the predicted *Trichoplax adhaerens* protein (JGI) but only Spag8 is present in the 26,069 *Trichoplax* ESTs (JGI and GenBank). By means of RACE (see [29] for protocols) we cloned four of these potential sperm markers (*spag8*, *dnajb13*, *mns1* and *meig1*) from Placozoa sp. H2, the lineage used here for the description of ultrastructural features. We were unable to amplify *nme5* in this lineage. Amplification attempts in the Placozoa sp. H16 using degenerate primers based on the *Trichoplax* and Placozoa sp. H4 sequence yielded no results even at low stringency conditions. The identified sperm-associated proteins group within five distinct categories representing different functions in vertebrates: category I protein Spag8 is related to sperm-oocyte recognition; the category II protein Dnajb13 is sperm-flagellum associated; category III and IV proteins Mns1 and Meig1 are involved in male gametocyte meiosis and spermatogenesis control, respectively, and the category V protein Nme5 functions in oxidative stress protection during spermatogenesis in the mouse model (see Table 1; [30]–[42]). All sperm-associated proteins show a Blast E-value below 1e-10 in Blastp against mouse RefSeq proteins (GenBank), which was set as a minimum cut off value in the reciprocal Blast searches. Three of the five putative sperm markers (Spag8, Mns1 and Meig1) revealed hits to homologous proteins from other taxa when blasted against the RefSeq database (GenBank) using a stringent cutoff value of 1e-20. The other two proteins, Dnajb13 and Nme5, belong to large gene super-families. We therefore assigned gene orthology using phylogenetic reconstructions and included sequences from other super-family members as well as sequences from the anthozoan *Nematostella vectensis* as another non-bilaterian representative (see Dataset S1 for accession numbers and Dataset S2 for alignments of placozoan and anthozoan Dnaj and Nme domains with orthologous and paralogous domains from other Metazoa). The phylogenetic analyses strongly support a grouping of the *Trichoplax* DnaJ and Nme proteins to homologs of the Dnajb13 and Nme5 gene families, respectively (Figure S1).

**Table 1:** Expressed placozoan homologs of mouse male germline markers.

gene name	category	*T. adhaerens* (H1) accession numbers (JGI-ID)	Placozoa sp. H2 accession numbers	Placozoa sp. H4 accession numbers (EST per cluster)	e-value of best hit against Genbank	e-value of best hit against mouse RefSeq proteins	location in mouse	function in mouse	references
sperm associated antigen 8 (*spag8*)	I	XP_002110904 ^a^	HM243486	HM243490 (2)	1e-27	2e-07	sperm acrosome	sperm-oocyte recognition and cell division during spermatogenesis	[30]–[32]
Spermatogenesis apoptosis-related protein (*dnajb13*)	II	XP_002112903	HM243487	HM243491 (1)	1e-113	5e-102	in cytoplasm of spermatids and associated with the axoneme of sperm flagellum	assembly and stability of axoneme during sperm flagellum development and assembly of the annulus structure	[33]–[36]
meiosis-specific nuclear structural protein 1 (*mns1*)	III	XP_002111307	HM243488	HM243492 (1)	8e-122	4e-84	pachytene stage during spermato-genesis	determination and maintenance of the appropriate nuclear morphology during meiotic prophase	[37], [38]
meiosis expressed gene 1 (*meig1*)	III, IV	XP_002109786	HM243489	HM243493 (1)	2e-18	7e-17	spermatocytes when initiating meiosis;	chromatin organization; key in the regulation of spermiogenesis	[39]–[41]
non-metastatic cells 5 (*nme5*)	V	XP_002112439	n.d.	HM243494 (1)	2e-69	2e-61	stage 12–16 spermatids	protection of developing male germ cells from beeing killed by oxidastive stress	[42]

Five homologs of mouse sperm-associated proteins – indicated by high E-values in blast searches – are active in adult, non-degenerating placozoan animals. These proteins were detected after screening EST sequences from Placozoa sp. H4 and subsequently retrieved from the *Trichoplax* genome (JGI) by blast searches and amplified from a Placozoa sp. H2 cDNA library (see Materials and Methods for details). The putative sperm markers fall within five distinct functional categories: category I = sperm-oocyte recognition; category II = sperm flagellum-associated, category III = male gametocyte meiosis; category IV = control of spermatogenesis; category V = oxidative stress protection. a = EST supported (JGI); n.d. = not detected. For all blast searches the predicted *Trichoplax adhaerens* protein sequences were used.

### Cell counting in developing embryos

**Figure 2:**
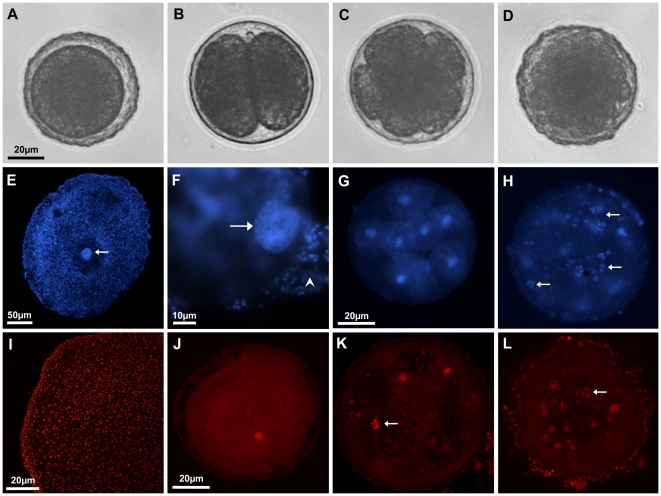
Cell counting in Placozoa sp. H2 embryos. Shown are embryos at the zygote-, 2-cell, 8-cell and 64-cell stage inside the fertilization membrane under light microscopy (A–D). Cleavage is total and equal. Nuclear staining with DAPI shows a direct correlation of blastomer number and fluorescent signals under standard fluorescent microscopy (F–H; 1, 8 and 64 cells, respectively). The same was seen with propidium iodide staining in confocal laser scanning images (J–L; 1, 8 and 120 cells, respectively). Red signals at the surface of the fertilization membrane in K and L derive from attached bacteria and algae to the fertilization membrane of the free drifting embryos. Positive controls for the staining procedure with adult animals showed clear nuclear signals for both fluorescent dyes (E, I). Maturing oocytes and zygotes show a large nucleus compared to somatic cells of the mother animal (arrow in E; F). (F) shows a zygote with a large, regular shaped interphase nucleus (arrow). Note that cells from the mother animal are still attached to the embryo (arrowhead). Metaphase chromosome clumps were regularly found in fluorescent staining, indicating normal cell cycle (arrow in H, K and L; compare Figure 3d2). The scale bars of A, G and J also apply to B–D, H and K–L, respectively.

To follow embryonic development beyond the 64-cell stage and to test the assumption that the cell cycle is disrupted at a very early stage of embryonic development, complete embryos were stained with nucleic acid intercalating fluorescent dyes. DAPI staining was initially used to identify nuclei in early embryos by means of standard fluorescence microscopy. The resulting DAPI signals were identical to the number of counted blastomers (Figure 2F–H). The above procedure allowed seeing nuclei as well as metaphase chromosomes in single blastomers (arrows in Figure 2H). All chromosomes were interconnected and thus found in distinct patches (compare [43]). To further count nuclei in later embryos, propidium iodide was used to stain nuclei. Confocal laser scanning microscopy revealed similar results as the DAPI staining, showing nuclei and metaphase chromosomes unambiguously labeled (Figure 2J–L). By counting the signal in all planes, a maximum of 120 cells were counted in Placozoa sp. H2. As cleavage of blastomers often occurs asynchronously this indicates the nearly complete128-cell stage. All embryos died after the observed 128-cell stage.

As a by-product numerous endosymbiontic bacteria were simultaneously stained and found in distinct patches both in oocytes and embryos (Figure S2).

### Ultrastructural analyses of developing oocytes and embryos

**Figure 3:**
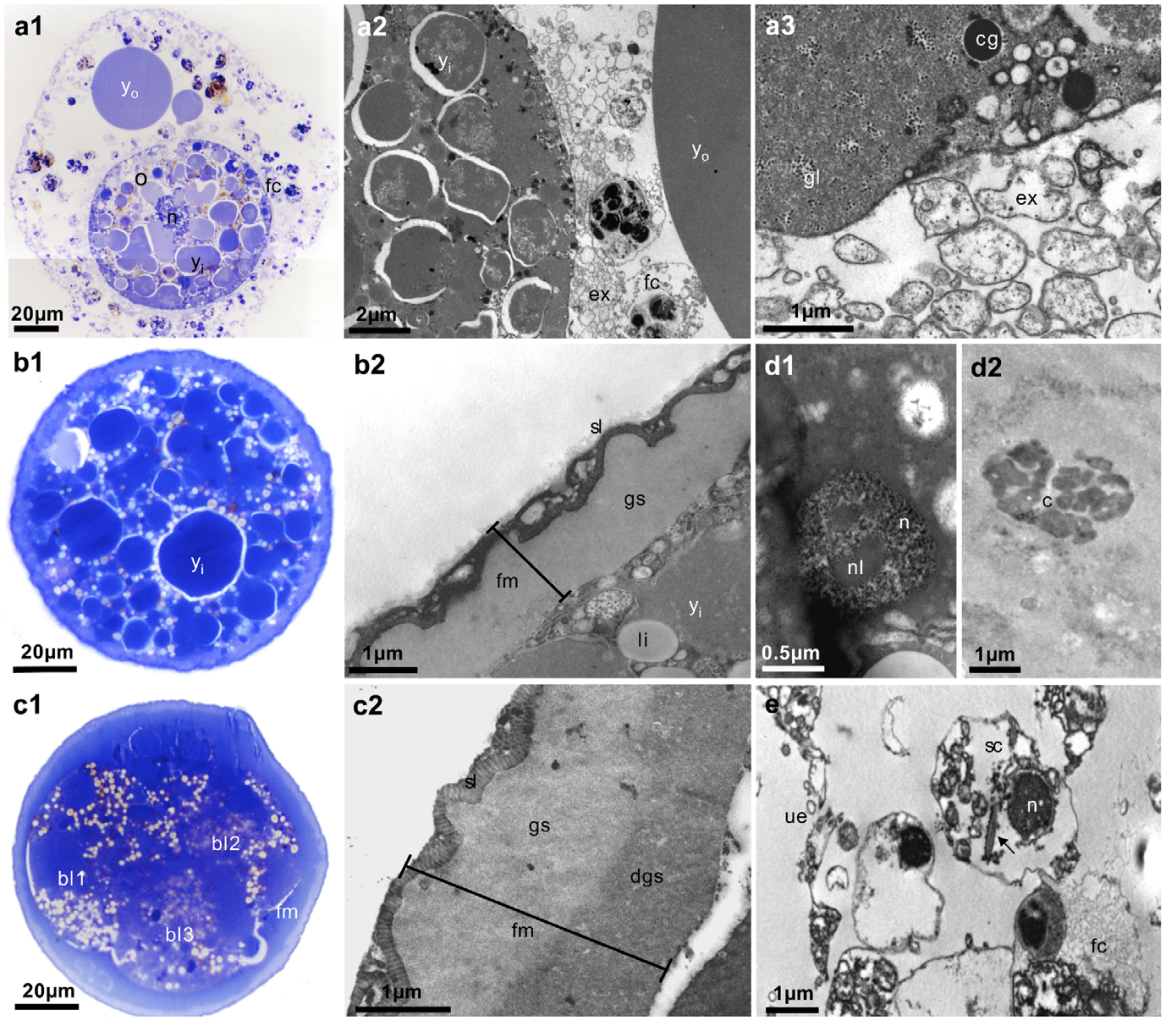
Ultrastructural analyzes of developing Placozoa sp. H2 oocytes and embryos. Shown are toluidine stained semi-thin sections (left panels) and TEM images (middle and right panels) of maturing oocytes (a) and embryos in different stages (b, c, d). Yolk material inside and outside maturing oocytes and embryos is clearly visible in dark blue in toluidine stained sections (a1, b1, c1) and as moderately electron dense material in TEM images (a2, b2). The early ‘fertilization membrane’ is made up of two layers (b1, b2), whereas three layers are distinguishable in later stages (c1, c2). Additional features not reported before are glycogen granules (a3) and lipid droplets in the oocyte (b1, b2, c1). In some sections nuclei (d1) and chromosomes (d2) were found in blastomers, indicating a normal cell cycle. We identified a putative sperm cell (e) with a retracted flagellum (arrow in e). o = oocyte, y_o_ = yolk outside oocyte, y_i_ = yolk inside oocyte, fc = fiber cell, ex = fiber cell extensions, cg = cortex granulum, gl = glycogen, li = lipid droplet, fm = fertilization membrane, sl = striped layer, gs = ground substance, dgs = dense ground substance, bl = blastomer, n = nucleus, nl = nucleolus, c = metaphase chromosomes, sc = putative sperm cell, ue = upper epithelium.

By means of toluidine staining and transmission electron microscopy, features of maturing placozoan oocytes and developing embryos known from *Trichoplax adhaerens* were studied in Placozoa sp. H2. All oocytes had a large nucleus with a diameter of close to 20 µm (Figure 3a1). Several fiber cells were always seen in close contact to the oocyte (Figure 3a2). These are distinguishable from other cell types by their characteristic mitochondrial complexes and concrement vacuoles [17], [44]. Extensions of these cells are absorbed by the oocyte, also allowing bacteria to be actively transferred. Cortical granules were found throughout the body of young oocytes, which migrate to the margin when the oocytes are mature (Figure 3a2, a3) (cf. [20]).

In addition to these formerly seen characteristics, several new features were found in Placozoa sp. H2 oocytes and embryos. The large droplets outside the oocyte were formerly described as ‘lipid droplets’ [17]–[19]. Our Toluidin and TEM images, however, suggest that these so-called ‘lipid droplets’ are actually made of yolk, they show the identical structure of yolk droplets seen inside the oocytes (Figure 3a1, a2). Lipid droplets are thus only found inside the oocytes (Figure 3b2) while yolk is also accumulated in the mother animal. Another feature newly found inside placozoan oocytes and embryos were glycogen granules and lipid droplets (Figure 3a3, b1, c1). Although not unusual for oocytes and embryos, these materials have not previously been recognized in *Trichoplax adhaerens*.

In early stages the two-layer structure of the fertilization membrane is made up of the ‘striped layer’ and the ‘ground substance’ (Figure 3b1, b2), comparable to the *Trichoplax* fertilization membrane. However, in embryos from the 4-cell stage onward a third layer was detected (Figure 3c1, c2). According to the structure and position under the ‘ground substance’, we refer to this layer as ‘dense ground substance’. Additionally, as observed in fluorescent staining, nuclei and metaphase chromosomes were visible in TEM sections (Figure 3d1, d2). The latter is another new feature for placozoans.

## Discussion

The Placozoa is one of the key phyla for unraveling early metazoan evolution. All morphological and several molecular traits indicate a basal position in the metazoan tree of life with the exact phylogenetic position being heavily discussed (e.g [5]). The only placozoan species described yet, *Trichoplax adhaerens*, has attracted substantial attention lately, and its genome was sequenced before the life cycle was known. We have provided evidence here that the life cycle includes bisexual reproduction and that different placozoan lineages differ in embryonic development.

Sexual reproduction can regularly be induced – as seen by oocyte maturation and early embryonic development – at least in three placozoan species lineages: *Trichoplax adhaerens* (the so-called ‘GRELL’ clone), the Placozoa sp. H2 (‘CAR-PAN-4’ clone) and Placozoa sp. H16 (‘KEN-A’ clone). One critical element for the induction of sexual reproduction is the temperature. In the test lineages described here production of oocytes only occurs at 23°C or above indicating a seasonal temperature-related component in the sexual life cycle of placozoans, similar to other basal metazoan animals like sponges (e.g. [45]–[49]) and cnidarians (e.g. [50]–[55]. We never found a single sexually reproducing animal at 22°C or below indicating a sharp temperature barrier between the vegetative and sexual mode of reproduction. According to this temperature-dependent onset of at least oocyte maturation we may expect in lineages from locations where the water temperature never drops below 25°C (here Panama [H2] and Kenya [H16]) to see either a year-round sexual reproduction, or other and/or additional factors triggering sexual reproduction in the natural habitat. Thus for researchers trying to develop placozoans as a model system for EvoDevo research the choice of the right placozoan species may be crucial.

Oocyte maturation and early cleavage stages of Placozoa sp. H2 resemble that of *Trichoplax adhaerens* described earlier [17], [18]. We were able to follow embryonic development beyond the 64-cell stage, but we could not complete the life cycle. Obviously some critical environmental factors, necessary for the completion of embryonic development, remain unknown. Since embryos beyond the 64-cell stage are released from the degenerating mother, and since they are not attached to the surface any longer, they enter a new ecological niche, the plankton. Possibly these new conditions are critical for further development. Subsequent studies need to identify these conditions in the laboratory, which will not be an easy task, however. Simple factors that can be tested include temperature and light exposure. More likely, however, the unknown conditions include a change in water quality, which offers a broad spectrum of factors to be investigated. We also suggest that crossing of different clonal lineages might increase the chances to complete the life cycle under laboratory conditions.

EST data from several species lineages show that the putative sperm marker genes are expressed at some point in adult animals. This observation is consistent with the hypothesis of bisexual reproduction (male and female gametes).

The potential sperm markers in three different placozoan representatives (*Trichoplax adhaerens*, Placozoa sp. H2 and Placozoa sp. H4) cover various stages of spermatogenesis ranging from early meiosis to sperm maturation, with functional flagella and sperm-oocyte recognition proteins used in fertilization. All markers were expressed in adult, healthy growing animals with no signs of degradation. This is true at least for Placozoa sp. H2 and Placozoa sp. H4 where cDNA was used to amplify these genes. Interestingly we were unable to isolate any of the five putative sperm markers from Placozoa sp. H16, which likely mirrors the strong sequence divergence between different placozoan lineages [25], [26], [56], [57] rather than a lack of spermatogenesis in sp. H16.

Phylogenetic analyses of Dnajb13 and Nme5 with related proteins from the DnaJ and Nme super-families unambiguously show that these proteins are homologs of mouse proteins known to play important roles in spermatogenesis at least in the mouse. The other three putative placozoan sperm markers Spag8, Mns1, and Meig1, however, do not belong to any gene family. Blast searches hit the following proteins only: Spag8, Mns1 and Meig1 (with E-values between 2e-18 to 8e-122). The mouse Spag8 is one out of eleven “sperm-associated antigens” entered in the Mouse Genome Informatics database (at http://www.informatics.jax.org/). Spag proteins, however, do not form a genealogical gene super-family and the artificial grouping is based only on their function in spermatogenesis. Thus, in the absence of sister proteins, the different mouse Spag proteins, which vary in length between 90–2320 amino acids, cannot be subjected to phylogenetic analysis. The same is true for Mns1 and Meig1. No related sister proteins exist that would allow phylogenetic orthology assignment. Thus, we conclude from similarity only that the placozoan Spag8, Mns1 and Meig1 proteins are homologs of the corresponding proteins in other species.

Transcription of the sperm-oocyte recognition marker *spag8* is linked to late or final stages of sperm in other animals. Although we have no functional data of *spag8* in placozoans, the homology to other *spag8* genes fuels the speculation of the production and storage of sperm during normal growth in the Placozoa. The latter seems to be the normal case for most bisexually reproducing animals, at least when they are dioecious [58]. The storage of sperm allows a more rapid sexual response to a changing environment for example. The laboratory animals start to degrade when conditions become sub-optimal. Degrading animals reduce their lower epithelium and stop feeding. As a result the energy for growing oocytes comes from the consumption of stored reserve materials in the animal's body. In this context producing sperm and oocytes consecutively or using different genders (i.e. being dioecious) would be advantageous.

With one exception we were not able to detect cells that fit the morphological description of sperm cells by Grell & Benwitz (1981) [12]. Only in Placozoa sp. H4 we found a flagellated putative sperm cell (Figure 3e). The identification of the putative sperm marker Dnajb13, which is associated with the sperm flagellum, indicates flagellated sperm in the Placozoa, a presumed ancestral feature of metazoans [59]. The observation that we found a single sperm cell in >1000 TEM section from more than 200 healthy growing as well as degrading animals might suggest that only a few individuals in a population produce sperm at low amounts.

Even in the absence of functional data for the identified sperm-associated proteins in placozoans some lines of arguments support their role in spermatogenesis. The expression of a sperm associated antigen (Spag) in the known regions of gametogenesis in a sponge [60], for example, suggests a highly conserved function of Spag proteins throughout the Metazoa. Support for this hypothesis has to come from functional studies.

Besides the function in spermatogenesis one has to note, however, that all mouse sperm-associated proteins, except *meig1*, are also weakly expressed in other tissues [32], [36], [61]–[63]. *Meig1* is only expressed in the testis in mammals. It will be interesting to unravel its function in Placozoa and Porifera and to elucidate if *meig1* expressing sperm cells are an ancestral feature of the Metazoa.

We have found cortical granules in oocytes that have been known from oocytes across different metazoan phyla [64]–[73]. These granules are a key element for building the cortex or fertilization membrane of a fertilized oocyte. The membrane protects the embryo from its environment and prevents polyspermy (e.g. [74], [75]). Like in other animals these cortical granules are evenly dispersed throughout early oocytes and later move towards the margins during maturation ([20]; own data). The granules disappear once the fertilization membrane is built, which supports the view of a participation in the generation of the protective eggshell after fertilization.

Another new feature is the three-layered fertilization membrane in Placozoa sp. H2, while the one in *Trichoplax adhaerens* is two-layered [20]. Subsequent studies must show whether this is a unique morphological character of one placozoan species-lineage or if a three-layered fertilization membrane can be found in other lineages as well. It must also be noted that only our studies discovered lipid droplets and glycogen in oocytes, features that were not observed before. We identified the ‘droplets’ that are seen in degenerating animals as yolk droplets. These look identical in coloration, density and structure to yolk droplets inside the oocyte and thus we name these ‘outer yolk droplets’ according to their occurrence outside the oocyte.

We have reported several new placozoan features and substantial evidence for recent bisexual reproduction in the Placozoa. Future studies will also be challenged by comparing developmental features between a yet unknown number of placozoan taxa. All of the above is of crucial importance for the steadily increasing number of developmental genetic studies that want to use *Trichoplax* as a basal metazoan model system [14].

## Materials and Methods

### Animal material and culture conditions

To study placozoan embryonic development four previously established clonal placozoan cultures were used: *Trichoplax adhaerens* (‘GRELL’ clone from Israel), Placozoa sp. H2 (‘CAR-PAN-4’ clone from Panama; 16S Haplotype H2), Placozoa sp. H4 (‘HWH-B’ clone from Hawaii) and Placozoa sp. H16 (‘KEN-A’ clone from Kenya) [11], [25], [26], [76]. These clones regularly reproduce sexually in our laboratory under formerly described standard conditions ([77], [78]; and see Text S1 for details on culturing). Unfortunately no study exists that addresses the effect of water chemistry.

### Identification of sperm-associated proteins in three placozoan species-lineages

In order to search for sperm-associated proteins we started with EST data from Placozoa sp. H4 (‘HWH-B’ clone, E. Gaidos, Hawaii), which can be grown in large quantities. This lineage is genetically distantly related to *Trichoplax adhaerens* (H1 lineage) and to the Placozoa sp. H2 lineage [11], [25], [26]. We generated 4,015 EST that were assembled resulting in 2,196 Placozoa sp. H4 transcript clusters (see Text S1 for details).

A reciprocal Blast-based search for sperm-associated proteins was performed on the newly generated EST data as well as on available data for *Trichoplax adhaerens* (for details see Text S1). Using this strategy we identified homologs of five sperm-associated candidate proteins in *Trichoplax adhaerens* and Placozoa sp. H4 using the JGI Blast server (http://genome.jgi-psf.org/Triad1/Triad1.home.html). In order to isolate these genes from Placozoa sp. H2, on which ultrastructural analyses on sexual reproduction were carried out, a cDNA library was constructed using RNA isolation methods as mentioned above. The cDNA was generated with the GeneRacer kit (Invitrogen). To amplify nearly complete coding sequences 3′-RACE was performed according to manufacturer's recommendations (Invitrogen) using 5′ genes-specific primers based on the *T. adhaerens* and Placozoa sp. H4 sequences, and the GeneRacer 3′ primers (the complete list of primers is available upon request).

### Cell counting by fluorescent DNA labelling

Zygotes with a ‘fertilization’ membrane as well as older developmental stages were isolated from D-phase animals. Embryos were fixed in sterile plastic six-well plates with 4% paraformaldehyde in artificial seawater. After fixation, embryos were washed for 5 minutes in 1× PBST (phosphate buffered saline; 0.1% Tween) before subsequent fluorescent staining using DAPI or propidium iodide (both Roche). For propidium iodide staining RNA was digested with 20 mg/ml RNase A (Roche) in 1× PBST for 30 minutes at 30°C to prevent background. The DNA was stained for one minute at room temperature in 1×PBS containing either 1× DAPI or 1× propidium iodide dissolved from 1000× stock solutions (2 mg/ml and 0.5 mg/ml stock concentrations, respectively). All steps were done in sterile plastic six-well plates. After staining, embryos were washed with 1× PBS, mounted on microscopic slides, and subsequently examined. Visualization was done on a Zeiss Axiovert 200 M fluorescence microscope (DAPI) and on Leica TCS SP2 confocal laser scanning microscope (PI). PI stained embryos were scanned and photographs were taken at 1 µm steps to follow single nuclei throughout the embryo and to prevent double counting.

### Scanning and transmission electron microscopy and toluidine blue staining

Eggs were isolated six weeks after starting new mass cultures. For TEM analysis eggs were fixed overnight in a 0.1 M phosphate buffered (pH 7.3) solution of paraformaldehyde (2%), glutaraldehyde (3%) and picric acid (7.5%) [76]. After washing in 0.1 M phosphate buffered (pH 7.3) solution (PBS), samples were post-fixed in 2% osmium tetroxide solution in the same buffer and rinsed in PBS again. Following dehydration in a graded acetone series samples were embedded in Araldite. Ultrathin sections were cut with a LKB Ultrotome 2088 V, double contrasted with alcoholic uranyl acetate and lead citrate, and observed under a Philips CM10 transmission electron microscope. Several 1 µm semithin sections were stained with toluidine blue and observed under an Olympus Vanox optical microscope. For SEM, after the post-fixation in osmium, samples were rinsed in PBS, dehydrated through a graded ethanol series and critical point-dried under CO_2_ atmosphere. After mounting on aluminum stubs, the samples were sputter coated with gold-palladium and observed with a Philips 515 scanning electron microscope.

## Supporting Information

Figure S1 **Neighbor Joining trees (BioNJ) of DnaJ (A) and Nme (B) protein domains.** The *Trichoplax adhaerens* Dnajb13 and Nme5 proteins clearly group to corresponding known family subgroups (green branches). *Trichoplax adhaerens* and *Nematostella vectensis* proteins are marked with blue and red branches, respectively.
(PDF)

Figure S2 **Endosymbiotic bacteria in Placozoa sp. H2 oocytes.** Many bacteria were found in patches as shown in DAPI stained and propidium iodide stained oocytes (A and B, respectively) and in TEM images (C). The bacteria are actively transferred to the maturing oocyte by extensions of fiber cells (see main text and Fig. 2). b = bacteria.
(PDF)

Dataset S1 Accession numbers used for the phylogenetic analyses underlying Fig. S1.
(PDF)

Dataset S2 Alignments of C-terminal DnaJ domains (A) and NDK domains (B) underlying phylogentic inferences in Fig. S1.
(PDF)

Text S1 Supporting material and methods.
(DOC)
