# Supplementary material for: New Insights into Placozoan Sexual Reproduction and Development
Source: PLoS One. 2011 May 19;6(5):e19639. doi: 10.1371/journal.pone.0019639 (PMC3098260; doi:10.1371/journal.pone.0019639)

Figure S1. Neighbor Joining trees (BioNJ) of DnaJ (A) and Nme (B) protein domains.

The *Trichoplax adhaerens* Dnajb13 and Nme5 proteins clearly group to corresponding known family subgroups (green branches). *Trichoplax adhaerens* and *Nematostella vectensis* proteins are marked with blue and red branches, respectively.

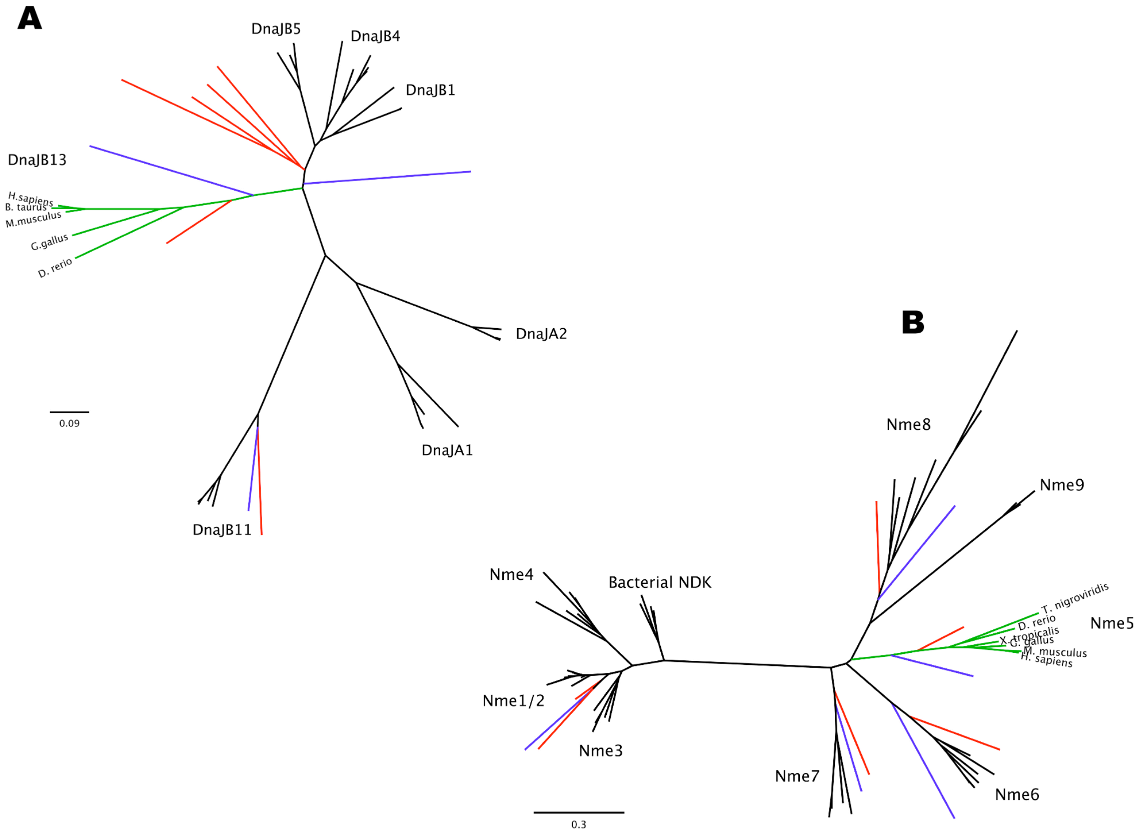

Supplement: Figure S1 — Neighbor Joining trees (BioNJ) of DnaJ (A) and Nme (B) protein domains. The Trichoplax adhaerens Dnajb13 and Nme5 proteins clearly group to corresponding known family subgroups (green branches). Trichoplax adhaerens and Nematostella vectensis proteins are marked with blue and red branches, respectively. (PDF) [file pone.0019639.s001.pdf]
