# Supplementary material for: New Insights into Placozoan Sexual Reproduction and Development
Source: PLoS One. 2011 May 19;6(5):e19639. doi: 10.1371/journal.pone.0019639 (PMC3098260; doi:10.1371/journal.pone.0019639)

Figure S2. Endosymbiotic bacteria in Placozoa sp. H2 oocytes.

Many bacteria were found in patches as shown in DAPI stained and propidium iodide stained oocytes (A and B, respectively) and in TEM images (C). The bacteria are actively transferred to the maturing oocyte by extensions of fiber cells (see main text and Fig. 2). b=bacteria.

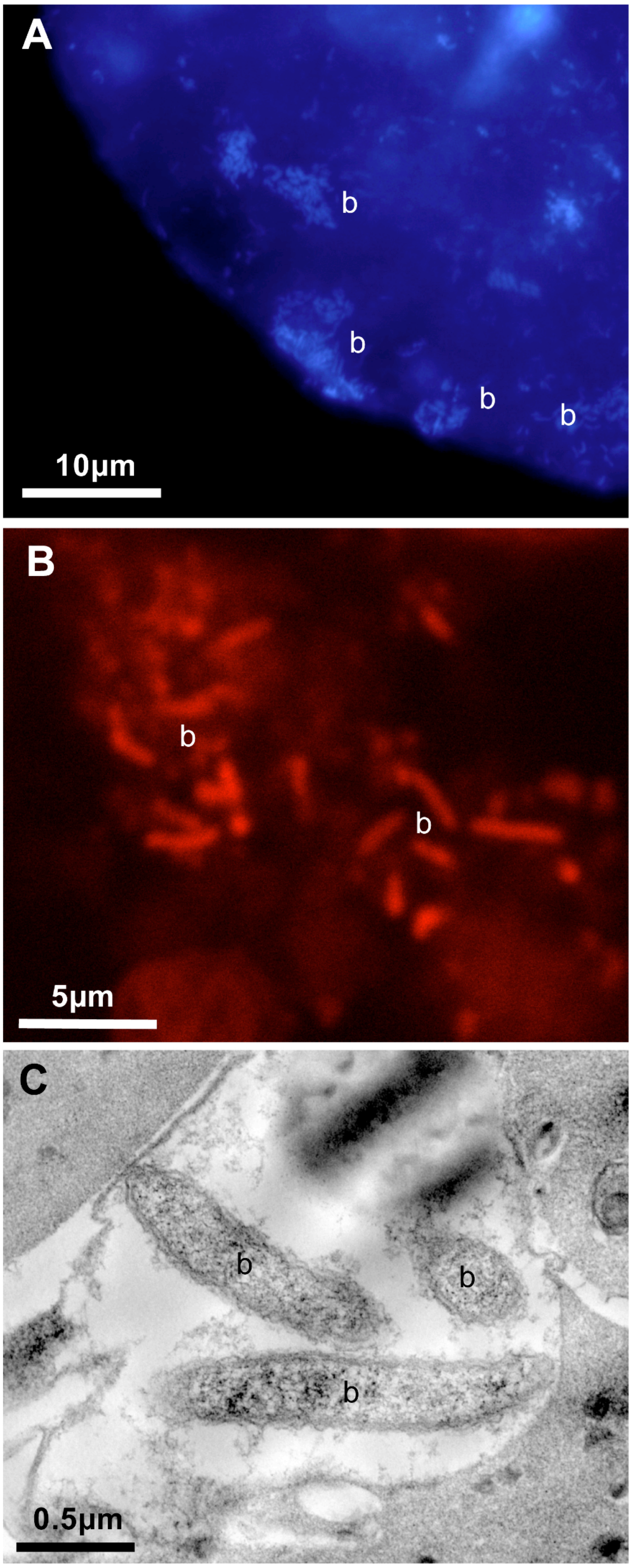

Supplement: Figure S2 — Endosymbiotic bacteria in Placozoa sp. H2 oocytes. Many bacteria were found in patches as shown in DAPI stained and propidium iodide stained oocytes (A and B, respectively) and in TEM images (C). The bacteria are actively transferred to the maturing oocyte by extensions of fiber cells (see main text and Fig. 2). b = bacteria. (PDF) [file pone.0019639.s002.pdf]
