# Supplementary material for: New Insights into Placozoan Sexual Reproduction and Development
Source: PLoS One. 2011 May 19;6(5):e19639. doi: 10.1371/journal.pone.0019639 (PMC3098260; doi:10.1371/journal.pone.0019639)
Supplement: Dataset S1 — Accession numbers used for the phylogenetic analyses underlying Fig. S1. (PDF) [file pone.0019639.s003.pdf]

Dataset S1. Accession numbers used for the phylogenetic analyses underlying Fig. S1.

protein	species	accession / JGI-ID
**DnaJA1**	*Homo sapiens*	NP_001530.1
	Bos taurus	NP_001015637.1
	Mus musculus	NP_032324.1
	Gallus gallus	NP_001012963.1
	Danio rerio	NP_955956.1
**DnaJA2**	*Homo sapiens*	NP_005871.1
	Bos taurus	NP_001035581.1
	Mus musculus	NP_062768.1
	Gallus gallus	NP_001005841.1
	Danio rerio	NP_998658.1
**DnaJB1**	*Homo sapiens*	NP_006136.1
	Bos taurus	NP_001028935.1
	Mus musculus	NP_061278.1
	Danio rerio	NP_956067.1
**DnaJB4**	*Homo sapiens*	NP_008965.2
	Bos taurus	NP_001039968.1
	Mus musculus	NP_080202.1
	Gallus gallus	XP_001233254.1
	Danio rerio	NP_001003455.1
**DnaJB5**	*Homo sapiens*	NP_001128477.1
	Bos taurus	NP_001014959.1
	Mus musculus	NP_063927.1
	Gallus gallus	XP_424983.2
	Danio rerio	NP_001093510.1
**DnaJB11**	*Homo sapiens*	NP_057390.1
	Bos taurus	NP_001029440.1
	Mus musculus	NP_080676.3
	Gallus gallus	XP_422682.1
	Danio rerio	NP_942116.1
**DnaJB13**	*Homo sapiens*	NP_705842.2
	Bos taurus	NP_001029708.1
	Mus musculus	NP_705755.2
	Gallus gallus	XP_417251.2
	Danio rerio	NP_001017606.1
**NDK**	*Cyanothece sp.*	YP_002484590.1
	Synechococcus sp.	YP_474521.1
	Acaryochloris marina	YP_001518040.1
	Thermosynechococcus elongatus	NP_681058.1
**Nme1**	*Homo sapiens*	NP_937818
	Mus musculus	NP_032730
	Bos taurus	NP_991387
	Monodelphis domestica	XP_001363771

**Nme2**	*Homo sapiens*	NP_001018149
	Mus musculus	NP_032731
	Bos taurus	NP_001069844
	Monodelphis domestica	XP_001363684
	Gallus gallus	NP_990378
	Xenopus tropicalis	NP_001005140
**Nme3**	*Homo sapiens*	NP_002504
	Mus musculus	NP_062704
	Xenopus tropicalis	NP_001005115
	Danio rerio	NP_571003
	Tetraodon nigroviridis	CAG02649
**Nme4**	*Homo sapiens*	NP_005000
	Mus musculus	NP_062705
	Gallus gallus	AAB99857
	Xenopus tropicalis	NP_001039239
	Danio rerio	NP_957489
	Tetraodon nigroviridis	CAG12673
**Nme5**	*Homo sapiens*	NP_003542
	Mus musculus	NP_542368
	Gallus gallus	XP_414687
	Xenopus tropicalis	NP_001072619
	Danio rerio	NP_001002516
	Tetraodon nigroviridis	CAG01205
**Nme6**	*Homo sapiens*	NP_005784
	Mus musculus	NP_061227
	Gallus gallus	XP_424474
	Xenopus tropicalis	NP_001123709
	Danio rerio	NP_571672
	Tetraodon nigroviridis	CAG09120
**Nme7**	*Homo sapiens*	NP_037462
	Mus musculus	NP_612187
	Xenopus tropicalis	NP_988903
	Danio rerio	NP_571004
**Nme8**	*Homo sapiens*	NP_057700
	Mus musculus	NP_853622
	Gallus gallus	XP_426021
	Xenopus tropicalis	NP_001121456
	Danio rerio	NP_001082944
	Tetraodon nigroviridis	CAG09297
**Nme9**	*Homo sapiens*	NP_835231
	Mus musculus	XP_893103
	Bos taurus	NP_001069083
**DnaJB1/4/5**	*Trichoplax adhaerens*	XP_002110157.1
**DnaJB11**	*Trichoplax adhaerens*	XP_002116502.1
**DnaJB13**	*Trichoplax adhaerens*	XP_002112903.1
**Nme1**	*Trichoplax adhaerens*	XP_002115688.1
**Nme5**	*Trichoplax adhaerens*	XP_002112439.1

**Nme6**	*Trichoplax adhaerens*	XP_002114036.1
**Nme7**	*Trichoplax adhaerens*	XP_002108466.1
**Nme8**	*Trichoplax adhaerens*	JGI-54375
**DnaJB1/4/5-a**	*Nematostella vectensis*	XP_001630804.1
**DnaJB1/4/5-b**	*Nematostella vectensis*	XP_001641423.1
**DnaJB1/4/5-c**	*Nematostella vectensis*	XP_001638784.1
**DnaJB1/4/5-d**	*Nematostella vectensis*	XP_001624003.1
**DnaJB11**	*Nematostella vectensis*	XP_001636828.1
**DnaJB13**	*Nematostella vectensis*	XP_001630748.1
**Nme2-a**	*Nematostella vectensis*	XP_001630017.1
**Nme2-b**	*Nematostella vectensis*	XP_001630018.1
**Nme5**	*Nematostella vectensis*	XP_001631264.1
**Nme6**	*Nematostella vectensis*	XP_001622626.1
**Nme7**	*Nematostella vectensis*	XP_001626602.1
**Nme8**	*Nematostella vectensis*	XP_001634297.1
