# Supplementary material for: New Insights into Placozoan Sexual Reproduction and Development
Source: PLoS One. 2011 May 19;6(5):e19639. doi: 10.1371/journal.pone.0019639 (PMC3098260; doi:10.1371/journal.pone.0019639)
Supplement: Dataset S2 — Alignments of C-terminal DnaJ domains (A) and NDK domains (B) underlying phylogentic inferences in Fig. S1. (PDF) [file pone.0019639.s004.pdf]

Dataset S2. Alignments of C-terminal DnaJ domains (A) and NDK domains (B) underlying phylogentic inferences in Fig. S1.

A

```
                   1                                                              64
Hs-DnajA1          TYYDVLGVKP NATQEELKKA YRKLALKYHP DKNPNEG--- -EKFKQISQA YEVLSDAKKR ELYD
Bt-DnajA1          TYYDVLGVKP NATQEELKKA YRKLALKYHP DKNPNEG--- -EKFKQISQA YEVLSDAKKR ELYD
Mm-DnajA1          TYYDVLGVKP NATQEELKKA YRKLALKYHP DKNPNEG--- -EKFKQISQA YEVLADSKKR ELYD
Gg-DnajA1          TYYDVLGVSP NASAEELKKA YRKLALKYHP DKNHNEG--- -EKFKQISQA YEVLSDPKKR DLYD
Dr-DnajA1          GFYDMLGVKP SASPEELKKA YRKLALKYHP DKNPTEG--- -EKFKQISQA YEVLSDAKKR EVYD
Hs-DnajA2          KLYDILGVPP GASENELKKA YRKLAKEYHP DKNPNAG--- -DKFKEISFA YEVLSNPEKR ELYD
Bt-DnajA2          KLYDILGVPP GASENELKKA YRKLAKEYHP DKNPNAG--- -DKFKEISFA YEVLSNPEKR ELYD
Mm-DnajA2          KLYDILGVPP GASENELKKA YRKLAKEYHP DKNPNAG--- -DKFKEISFA YEVLSNPEKR ELYD
Gg-DnajA2          KLYDILGVPP GASDNELKKA YRKLAKEYHP DKNPNAG--- -DKFKEISFA YEVLSNPEKR ELYD
Dr-DnajA2          KLYDILGVSP SASENELKKA YRKLAKEYHP DKNPNAG--- -DKFKEISFA YEVLTNPEKR DMYD
Hs-DnaJB1          DYYQTLGLAR GASDEEIKRA YRRQALRYHP DKNKEPG--A EEKFKEIAEA YDVLSDPRKR EIFD
Bt-DnaJB1          DYYQTLGLAR GASDEEIKRA YRRQALRYHP DKNKEPG--A EEKFKEIAEA YDVLSDPRKR EIFD
Mm-DnaJB1          DYYQTLGLAR GASDDEIKRA YRRQALRYHP DKNKEPG--A EEKFKEIAEA YDVLSDPRKR EIFD
Dr-DnaJB1          DYYSVLGIQK GASDDEIKKA YRKQALKYHP DKNKSAG--A EEKFKEIAEA YDVLSDPKKK DIYD
Hs-DnaJB4          DYYCILGIEK GASDEDIKKA YRKQALKFHP DKNKSPQ--A EEKFKEVAEA YEVLSDPKKR EIYD
Bt-DnaJB4          DYYCILGIEK GASDEDIKKA YRKQALRFHP DKNKSPQ--A EERFKEVAEA YEVLSDPKKR EIYD
Mm-DnaJB4          DYYHILGIDK GATDEDVKKA YRKQALKFHP DKNKSPQ--A EEKFKEVAEA YEVLSDPKKR EIYD
Gg-DnaJB4          DYYSILGIEK GASEEDIKKA YRKQALKWHP DKNKSAH--A EEKFKEIAEA YEVLSDPKKR DIYD
Dr-DnaJB4          DYYKILGITK GASDDDIKKA YRKQALKWHP DKNKAAN--A EEKFKEVAEA YEVLSDPKKR EIYD
Hs-DnaJB5          DYYKILGIPS GANEDEIKKA YRKMALKYHP DKNKEPN--A EEKFKEIAEA YDVLSDPKKR GLYD
Bt-DnaJB5          DYYKILGIPS GANEDEIKKA YRKMALKYHP DKNKEPN--A EEKFKEIAEA YDVLSDPKKR GLYD
Mm-DnaJB5          DYYKILGIPS GANEDEIKKA YRKMALKYHP DKNKEPN--A EEKFKEIAEA YDVLSDPKKR SLYD
Gg-DnaJB5          DYYKILGIQS GANEDEIKKA YRKMALKYHP DKNKDPN--A EEKFKEIAEA YDVLSDPKKR AVYD
Dr-DnaJB5          DYYKILGIPS GSNEDEIKKA YRKMALKFHP DKNKDPN--A EEKFKEIAEA YEVLSDPKKR VIYD
Hs-DnaJB11         DFYKILGVPR SASIKDIKKA YRKLALQLHP DRNPDDP-QA QEKFQDLGAA YEVLSDSEKR KQYD
Bt-DnaJB11         DFYKILGVPR SASIKDIKKA YRKLALQLHP DRNPDDP-RA QEKFQDLGAA YEVLSDSEKR KQYD
Mm-DnaJB11         DFYKILGVPR SASIKDIKKA YRKLALQLHP DRNPDDP-QA QEKFQDLGAA YEVLSDSEKR KQYD
Gg-DnaJB11         DFYKILGVSR GASVKDIKKA YRKLALQLHP DRNPDDP-RA QEKFQDLGAA YEVLSDEEKR KQYD
Dr-DnaJB11         DFYKILGVSR SASVKDIKKA YRKLALQLHP DRNQDDP-NA QDKFADLGAA YEVLSDEEKR KQYD
Hs-DnaJB13         DYYSVLGITR NSEDAQIKQA YRRLALKHHP LKSNEPS--S AEIFRQIAEA YDVLSDPMKR GIYD
Bt-DnaJB13         DYYSVLQITR NSEDAQIKNA YRKLALKNHP LRSIEPG--A VETFRQIAEA YDVLSDPVKR GIYD
Mm-DnaJB13         DYYAVLQVTR NSEDAQIKKA YRKLALKNHP LKSSEPG--A PEIFKQIAEA YDVLSDPVKR GIYD
Gg-DnaJB13         DYYAVLELGR NATDADIKKA YRLLALENHP QKCKEPL--A QERFRLLAEA YDVLSDPVRR GIYD
Dr-DnaJB13         DYYAILEINR NAIDADIKKA YRRLALKHHP RSNSHAR--A AERFNLLAEA FDVLSDPRKK ATYD
Ta-DnaJB1/4/5      DYYQILGVQH NATDDEIKKA YRKMALKYHP DKNKDKN--A EEIFKDVAEA YEVLSDKEKR GIYD
Ta-DnaJB11         DFYKILGVDR DATLKQVKKA YRKLAIKYHP DKNKDDP-KA QDKFQDINAA YEVLSDEEKR KTYD
Ta-DnaJB13         DYYKILQITQ NVKSQDIKKA YRKFALKYHP DRNTAID--A VDKFKEVSEA YDVLSNGIRR AIYD
Nv-DnaJB1/4/5-a    NYYAILGVPR NASDDDIKKA YRRQALIFHP DKNKNSG--A EEKFKEISEA YKVLTDPRQR DIFD
Nv-DnaJB1/4/5-b    DYYAVLNVDK AASADDIKKA YRKQALKYHP DKNKSPG--A EEKFKEISEA YEVLSDPKKK EIYD
Nv-DnaJB1/4/5-c    NYYDILGVKK DASDQELKKA YKKQAFKYHP DKNKDPG--A EEKFKEIAEA YEVLSDPQKR EIFD
Nv-DnaJB1/4/5-d    NYYEVLGVER NATTDDIRRA YRRLALKYHP DKNAGTE--- -ENFKEVSEA YEVLCDPQQR ERFD
Nv-DnaJB11         DFYAILGVPR DASKNQIKRA YRKLAMKLHP DKNKDDP-KA QEKFHDIGAA YEVLADDDQR KIYD
Nv-DnaJB13         DYYDILGLTR SATDADIKKE YRKLSLKYHP DKNQEPS--A EVKFRQAAEA YDVLSDPKKR AIYN
```

B

```
             1                                                                                80
Csp-NDK      ERTFLAVKPD GVQRALV--- ---------- GEIIRRFEAK GFKLVGLKLM NVSKDLAEQH YGEHKEKPFF PGLVQFITSG
Ssp-NDK      ERTFIAIKPD GVQRGLV--- ---------- GSIIQRLESR GYQLVGLKLV QVSQELAEAH YAEHRERPFF PGLVKFITSG
Am-NDK       ERTFLAVKPD GVQRGLV--- ---------- GEIISRYEAK GFTLVGLKLM VVSRELAEQH YGEHKEKPFF SGLVDFITSG
Te-NDK       ERTFLAIKPD GVQRGLV--- ---------- GTIIQRFEQK GYTLVGLKLM RVSRELAEQH YGEHKDKPFF PGLVNFITSG
Hs-Nme1      ERTFIAIKPD GVQRGLV--- ---------- GEIIKRFEQK GFRLVGLKFM QASEDLLKEH YVDLKDRPFF AGLVKYMHSG
Mm-Nme1      ERTFIAIKPD GVQRGLV--- ---------- GEIIKRFEQK GFRLVGLKFL QASEDLLKEH YTDLKDRPFF TGLVKYMHSG
Bt-Nme1      ERTFIAIKPD GVQRGLM--- ---------- GEIIKRFEQK GFRLVAMKFM RASEDLLKEH YIDLKDRPFF AGLVKYMHSG
Md-Nme1      ERTFIAIKPD GVQRGLI--- ---------- GEIVKRFEQK GFHLVALKFM QASEDLLREH YIDLKDRPFY AGLVKYMHSG
Hs-Nme2      ERTFIAIKPD GVQRGLV--- ---------- GEIIKRFEQK GFRLVAMKFL RASEEHLKQH YIDLKDRPFF PGLVKYMNSG
Mm-Nme2      ERTFIAIKPD GVQRGLV--- ---------- GEIIKRFEQK GFRLVAMKFL RASEEHLKQH YIDLKDRPFF PGLVKYMNSG
Bt-Nme2      ERTFIAIKPD GVQRGLV--- ---------- GEIIKRFEQK GFRLVAMKFL QASEELLKQH YIDLKDRPFF PGLVKYMNSG
Md-Nme2      ERTFIAIKPD GVQRGLV--- ---------- GDIIKRFEQK G-SLVAMKFL RASEEHLKQH YVDLKDRPFF PGLVKYMNSG
Gg-Nme2      ERTFIAIKPD GVQRGLV--- ---------- GEIIKRFEQK GFRLVAMKFV HASEDLLKQH YIDLKDRPFY PGLVKYMNSG
Xt-Nme2      ERTFIAIKPD GVQRGLM--- ---------- GEIIKRFEQK GFYLVAMKFV QASKDLLKQH YIDLKDRPFY PGLVDYMSSG
Hs-Nme3      ERTFLAVKPD GVQRRLV--- ---------- GEIVRRFERK GFKLVALKLV QASEELLREH YAELRERPFY GRLVKYMASG
Mm-Nme3      ERTFLAVKPD GVQRRLV--- ---------- GEIVRRFERK GFKLVALKLV QASEELLREH YVELREKPFY SRLVKYMSSG
Xt-Nme3      ERTFLAIKPD GYQRRLI--- ---------- GEIIRRFEKK GFHLVAMKIM QASEQLLKQH YIALQDKPFY DRLVKYMGSG
Dr-Nme3      ERTFLAVKPD GVQRRLV--- ---------- GEIIRRFERK GFKLVGMKLL QASEAQLRQH YWELREKPFY NGLVKYMSSG
Tn-Nme3      ERTFIALKPD GVQRKLV--- ---------- GEIVRRFEKK GFKLVALKLV QAPQDLLRKH YSDLSRRPFF GELVRYMSSG
Hs-Nme4      ERTLVAVKPD GVQRRLV--- ---------- GDVIQRFERR GFTLVGMKML QAPESVLAEH YQDLRRKPFY PALIRYMSSG
Mm-Nme4      ERTLVAVKPD GVQRRLV--- ---------- GTVIQRFERR GFKLVGMKML QAPESILAEH YRDLQRKPFY PALISYMSSG
Gg-Nme4      EQTLVLVKPD AVQRRLV--- ---------- GDVIGRFERR GFKLVAMKLL QADRGLLDRH YQHLQQKSFY PALLAYMTSG
Xt-Nme4      ERTLIAVKPD GVQRKLV--- ---------- GEIIKRFEQR GFTLVGLKLL QASEGILAEH YHDLRRKPFY PALLRYMASG
Dr-Nme4      ERTLVAVKPD GVQRRLI--- ---------- GEVIKRFEQR GFRLVGLKML QAPDKLLAQH YVSLQKKPFY SSLLYYMTSG
Tn-Nme4      ERTLIVVKPD GVQRRLV--- ---------- GRIIQRFEQR GFKMVGLKML QVSEDLLSNH YRQLRMKPFY SDLVQYMTSG
Hs-Nme5      EKTLAIIKPD IVDKE----- ---------- EEIQDIILRS GFTIVQRRKL RLSPEQCSNF YVEKYGKMFF PNLTAYMSSG
Mm-Nme5      EKTLALIKPD VVDKE----- ---------- EEIQDIILGS GFTIIQRRKL HLSPEHCSNF YVEQYGKMFF PNLTAYMSSG
Gg-Nme5      EKTLALIKPD VVAKE----- ---------- EEIEDLILRS GFMIVQKRKL QLSPEQCSIF YADQYGKMFF PNLAAYMSSG
Xt-Nme5      ERTLAIIKPD VLHKA----- ---------- EEIEDIILRC GFHIVQKRKV HLSPEQCSDF YSDQYGKMFF PSLTAYMSSG
Dr-Nme5      ERTLALIKPD AIHKT----- ---------- DEIEDIILQS GFTILQKRRL QLSPEQCSDF YAEHYGKLHF PHLTAFMSSG
Tn-Nme5      QQTLALIKPD VIHIA----- ---------- DEIEGEILKW GFFILQKRKL QLSPEHCSDF YADQYGTPHF PGLTAFMSSG
Hs-Nme6      -LTLALIKPD AVAHPLIL-- ---------- EAVHQQILSN KFLIVRMREL LWRKEDCQRF YREHEGRFFY QRLVEFMASG
Mm-Nme6      -LTLALIKPD AVAHPLIL-- ---------- EAVHQQILSN KFLIVRTREL QWKLEDCRRF YREHEGRFFY QRLVEFMTSG
Gg-Nme6      -LTLALLKPD AVAHPLVL-- ---------- EAVHETILSN RLLIVRAKEL RCGREQSRRF YREHAGQFFY QRLVEFMASG
Xt-Nme6      -LTLALIKPD AVANPVIS-- ---------- EAVHQKILEN NFLIIRHKEL HWRSTDSQRF YCEHKGRFFY QRLVEFMSSG
Dr-Nme6      -LTLAVIKPD AMAHPLIL-- ---------- EALHQKILEN -FIIIRKTDL IWRTADSEMF YAEHQGRSSF KRLVEFMSTG
Tn-Nme6      -LTLAVIKPD AVAHPLML-- ---------- QALHQRILDN SFAIVRCKDL VWRKQDSERF YAEHSGRFFY QRLVEFMSSG
Hs-Nme7A     EKTLALIKPD AISKA----- ---------- GEIIEIINKA GFTITKLKMM MLSRKEALDF HVDHQSRPFF NELIQFITTG
Mm-Nme7A     EKTLALIKPD AVSKA----- ---------- GEIIEMINKS GFTITKLRMM TLTRKEAADF HVDHHSRPFY NELIQFITSG
Xt-Nme7A     EKTLALIKPD AVTKM----- ---------- GSIIEAILDS GFVISKAKMV LLLRTEAMDF YNEHHSKSFF SDLISFMTSG
Dr-Nme7A     ERTLAMIKPD AVSKV----- ---------- GDIIQMIYDA NLIVTKAKMT KLTWKQAADF YMEHQSKSFF NNLVQFVSSG
Hs-Nme8B     EKTLALLRPN LFHERK---- ---------- DDVLRIIKDE DFKILEQRQV VLSEKEAQAL CKEYENEDYF NKLIENMTSG
Mm-Nme8B     QTTLALLHPD ICEEEK---- ---------- DDVLNVIHNE GFTILMQRQI VLSEEEARTV CKIHENEEYF DNLIGHMTSN
Gg-Nme8B     EKTLALIRPC VLRERR---- ---------- NSIMQSIKDD GFEVAMQKEI TLSEEQAREF YKEHENEDYF PALLEQMTSG
Xt-Nme8B     ERTLALIRPD ILKDKK---- ---------- DEILQSIRDA GFSIAMQKEV MLTEQQVQEF YIEHIDKDYY PALLKQMTSG
Dr-Nme8B     ERTLALVRPD AARENR---- ---------- EEILSRIRQA GFRVAMQKEL MLTEEQVRLF YSTHVEEEYF NSLMENMTSG
Tn-Nme8B     ERTLALIRPD VARESR---- ---------- EKILSQIKES GFTVALQREV LLTEEQVTQF YSQHLEEDCF PALLHAMTSG
Hs-Nme9      -CTLAIIKPD AVAHGKT--- ---------- DEIIMKIQEA GFEILTNEER TMTEAEVRLF YQHKAGEEAF EKLVHHMCSG
Mm-Nme9      --TLGIIKPD AVAHGKA--- ---------- EEIIMKIQEA GFDILLKEER TLTEAEMQAF YQHRAREEAF ERLVHHMCSG
Bt-Nme9      --TLAIIKPD AVVHGKT--- ---------- DEIIMKIQEA GFDILTNEER TMTEAEMRLF YQHRAGEDTF EKLVHHMCSG
Ta-Nme1      ERTYIMVKPD GVDRGLV--- ---------- GDIISRFEKR GYKLVALKLK LASEELLREH YADLAGKPFF NGLVAFIGSG
Ta-Nme5      QCTLAIIKPN AVRKE----- ---------- DEIVELLEKQ GFCVLQRRCV RLTSEQASEF YTEHYGKMFF PALVTFMSSG
Ta-Nme6      QATLAIFKPD IMLHPSRV-- ---------- QKIQQLIEKN GIKVFRKKPL TMDVNTAEKF YGEHQGKFFY PRLVNLMTGG
Ta-Nme7A     ERTYAMIKPD AFSKLGILQL LVYGLYNSKR GEIVDIIVAD GFKICNLKSI QLSRKEAAEF YSEHEGKHFF NTLLDFMTSG
Ta-Nme8B     QRTLALIRPE TDSESK---- ---------- QRIIGAIEDA GFTIAIQKEI QLTREEAEEF YREHKSKDYF EKLITRMTSG
Nv-Nme2-a    ERTFIMVKPD GVQRGLV--- ---------- GEIIKRFEQK GFKLVALKMV QESEEHLKKH YADLAHLPFY PGLVKFMSSG
Nv-Nme2-b    ERTFLMIKPD AVSRGLI--- ---------- GEIISRFEKK GFKLVAMKFV KKSEDHFRKH YESLAKLKFY DGLCKYMSQT
Nv-Nme5      ERTLALIKPD AVHKS----- ---------- DEIEEIILQH GFTILQKRRA HLTPEQTSDF YAEHYGKMFF PSLVAYMSSG
Nv-Nme6      QLTLAILKPD LMLHPVRT-- ---------- QAVKNILVDN QFMIVRSRVL KWSREDAECF YTEHKGRFFY NRLVGFMSSG
Nv-Nme7A     EKTLAMIKPD AVGCL----- ---------- GGIMEMIDQA GFKLCRAKMV RLNRKEASDF YQEHASQPFY DRLVEFISSG
Nv-Nme8B     QRTLALIRPD ALRSRR---- ---------- ESIMSKIQEA GFEIAMSKEM HLTREQAEEF YSEHKDQEFF DTLVTNMSSG
```

```
             81                                                                                                155
Csp-NDK      PVVAMVWEG- ---KGVVASA RKIIGATNPL NS---EPGTI RGDYGVDIG- RNIIHGSDAV ETAQREIALW FQPAE
Ssp-NDK      PVVAMVWQG- ---KGVIAAA RKLIGKTNPL DA---EPGTI RGDFGIDIG- RNLVHGSDGP ETAQREIALW FQESE
Am-NDK       PVVAMVWEG- ---KGVVAAA RKIIGATNPL GS---EPGTI RGDFGIDIG- RNIIHGSDAV ETAQREISLW FKSEE
Te-NDK       PVVAMVWEG- ---RGVIANA RKLIGATNPL NA---EPGTL RGDFAVDVG- RNVIHGSDSP ENAEREINLW FQTQE
Hs-Nme1      PVVAMVWEG- ---LNVVKTG RVMLGETNPA DS---KPGTI RGDFCIQVG- RNIIHGSDSV ESAEKEIGLW FHPEE
Mm-Nme1      PVVAMVWEG- ---LNVVKTG RVMLGETNPA DS---KPGTI RGDFCIQVG- RNIIHGSDSV KSAEKEISLW FQPEE
Bt-Nme1      PVVAMVWEG- ---LNVVKTG RVMLGETNPA DS---KPGTI RGDFCIQVG- RNIIHGSDSV ESAEKEIALW FHPEE
Md-Nme1      PVVAMVWEG- ---LNVVKTG RMMVGETNPA DS---KPGTV RGDFCIQSG- RNIIHGSDSV ESAEKEIGLW FHPNE
Hs-Nme2      PVVAMVWEG- ---LNVVKTG RVMLGETNPA DS---KPGTI RGDFCIQVG- RNIIHGSDSV KSAEKEISLW FKPEE
Mm-Nme2      PVVAMVWEG- ---LNVVKTG RVMLGETNPA DS---KPGTI RGDFCIQVG- RNIIHGSDSV ESAEKEIHLW FKPEE
Bt-Nme2      PVVAMVWEG- ---LNVVKTG RVMLGETNPA DS---KPGTI RGDFCIQVG- RNIIHGSDSV KSAEKEINLW FKPEE
Md-Nme2      PVVAMVWEG- ---LNVVKTG RVMLGETNPA DS---KPGTI RGDFCIQVG- RNIIHGSDSV KSAEKEISLW FKPEE
Gg-Nme2      PVVAMVWEG- ---LNVVKTG RVMLGETNPA DS---KPGTI RGDFCIQVG- RNIIHGSDSV ESAQKEISLW FKPAE
Xt-Nme2      PVLAMVWEG- ---LNVVKTG RVMLGETNPA DS---KPGTI RGDFCIQVG- RNIIHGSDSV ESANKEIALW FEDKE
Hs-Nme3      PVVAMVWQG- ---LDVVRTS RALIGATNPA DA---PPGTI RGDFCIEVG- KNLIHGSDSV ESARREIALW FRADE
Mm-Nme3      PVVAMVWQG- ---LDVVHAS RALIGATDPG DA---MPGTI RGDFCMEVG- KNVIHGSDSV ESAHREIALW FREAE
Xt-Nme3      PVVAMVWQG- ---LDVVKTA RLMIGETNPA HS---LPGTI RGDFCVDVG- RNVIHGSDSR ESAQREIALW FQPDE
Dr-Nme3      PIVAMVWQG- ---LDVVKTA RKMLGETNPA DS---LPGTI RGDYCVEVG- RNVIHGSDSV ESAAREISLW FEDHE
Tn-Nme3      PVVAMVWQG- ---QDVVKTA RKMLGETNPA DS---LPGTI RGDSCVDVG- RNVIHGSDSV ESAQKEIYLW FRPHE
Hs-Nme4      PVVAMVWEG- ---YNVVRAS RAMIGHTDSA EA---APGTI RGDFSVHIS- RNVIHASDSV EGAQREIQLW FQSSE
Mm-Nme4      PVVAMVWEG- ---PNVVHIS RAMIGHTDST EA---APGTI RGDFSVHIS- RNVIHASDSV DGAQREIELW FQSSE
Gg-Nme4      PLVAMVWEG- ---YNVVRST RAMVGDTDSA QA---AAGTI RGDLSMHVS- RNVVHASDSV ETALREIGFW FQRDE
Xt-Nme4      PVVAMVWEG- ---HNVVRTS RAMVGDTDSS QA---KPGTI RGDFSVHIS- RNVIHASDSV EVAEREISLW FHSGE
Dr-Nme4      PIVAMVWEG- ---HNVVKTS RMMVGDTDPA AA---APGTI RGDFSVHIS- RNVVHASDSV EGAQREISLW FHRSE
Tn-Nme4      PVVVMAWEG- ---HQVIQSS RNMVGQTNPA EA---QAGTV RGDFSLHVS- RNVVHASDSP EGALRELQLW FRGQE
Hs-Nme5      PLVAMILAR- ---HKAISYW LELLGPNNSL VAKETHPDSL RAIYGTDDL- RNALHGSNDF AAAEREIRFM F-PE-
Mm-Nme5      PLVAMILAR- ---HKAISYW KELMGPSNSL VAKETHPDSL RAIYGTDEL- RNALHGSNDF AASEREIRFM F-PA-
Gg-Nme5      PSVAMILAR- ---HRAVSYW KELLGPSNSI KARMTHPHSL RAIYGTDDL- RNGLHGSLST SSAEREIRFM F-PE-
Xt-Nme5      PIIAMTLAR- ---YNAISYW KELIGPTNSL KAKETHPESL RAIYGTDDL- RNALHGSYCF TSAEREIRFM F-PEA
Dr-Nme5      PVVALALAR- ---DQAIATW KAIMGPVSSI KARETHPDCL RARFGTCDL- RNAVHGSETF SAAEREIRFM F-PHS
Tn-Nme5      PIIAMVLSR- ---DDAISYW KDLIGPSNSV IAKKTHPDSL RAKYGTSEI- QNALHGSESL PASVREIKFM F-PNT
Hs-Nme6      PIRAYILAH- ---KDAIQLW RTLMGPTRVF RARHVAPDSI RGSFGLTDT- RNTTHGSDSV VSASREIAAF F-PD-
Mm-Nme6      PIRAYILAH- ---KDAIQLW RTLMGPTRVF RARYIAPDSI RGSLGLTDT- RNTTHGSDSV VSASREIAAF F-PD-
Gg-Nme6      PMWAYILAH- ---ENAISLW RSLMGPTKVF RARNCVPDSI RGAYGLTDT- RNTTHGSDSP ASASREIAFF F-PE-
Xt-Nme6      PMQAYILAH- ---EDAVQLW RNLMGPTKVF RARIVAPGTV RGDLGLTDT- RNTTHGSDSV ESACREITFF F-PEF
Dr-Nme6      QMRAYILAR- ---EDAITHW RTMMGPTKVF RARFSSPETL RGKYGLTDT- RNTTHGSDSI ESAKREISFF F-PE-
Tn-Nme6      PMRAYILAR- ---EDAIRHW RELMGPTKVF RARHTVPASI RAQFGLTDT- RNTTHGSDSV ESAQREICFF F-PE-
Hs-Nme7A     PIIAMEILR- ---DDAICEW KRLLGPANSG VARTDASESI RALFGTDGI- RNAAHGPDSF ASAAREMELF F-PS-
Mm-Nme7A     PVIAMEILR- ---DDAICEW KRLLGPANSG LSRTDAPGSI RALFGTDGV- RNAAHGPDTF ASAAREMELF F-PS-
Xt-Nme7A     PIVAMEVVG- ---DEAVSSW RKLLGPTNSS IARSELPQSI RARFGTDGT- KNAAHGSDSI ASAARELEFF F-PS-
Dr-Nme7A     PVIAMELMG- ---DEAVSTW RKVLGPTDSG VAQKEAAHSL RGQFGTDGT- KNAGHGSDSL ASAARELEYF F-PS-
Hs-Nme8B     PSLALVLLR- ---DNGLQYW KQLLGPRTVE EAIEYFPESL CAQFAMDSLP VNQLYGSDSL ETAEREIQHF F-P--
Mm-Nme8B     HSYVLALRR- ---ENGVEYW KTLIGPKTIE EAYASHPQSL CVQFASGNFP TNQFYGSSSK AAAEKEIAHF F-PPQ
Gg-Nme8B     PTLVLALTR- ---QNAIQHW RDLLGPKTIE EAK-KVPNSL RAKYAIDNIA INQLHGSSSV NDAQKELEFF F-PQE
Xt-Nme8B     PVLALALVK- ---DHAVDHW RNMLGPASLR QALSEAPDSL RAQFAPNDSD INQLHGSSTP EEAKKELNFF F-P--
Dr-Nme8B     LVLALALVK- ---EGAVEHW RNILGPKDPI KAKNEQPDSL RAQFSVENSS INQLHGSSSS EEAEKEISFF F-PPE
Tn-Nme8B     PVLALALAR- ---KEAVCHW RNMLGPSDVN KAKEEDPESL RAQFAVGSAS INQLHGSASH EEAEREIRFF F-PPQ
Hs-Nme9      PSHLLILTRT EGFEDVVTTW RTVMGPRDPN VARREQPESL RAQYGTEMP- FNAVHGSRDR EDADRELALL F-PS-
Mm-Nme9      PSHLLILTKT EGTEDVVTAW RTFLGPCDPN VARREHPESL RAQYGTEMP- FNAVHGSRDR EDANRELALL F-PSF
Bt-Nme9      PSHLLILART EGTEDVVTAW RTLMGPCDPH VARREQPDSL RAQYGTEMP- FNAVHGSWDS EDARRELALL F-PG-
Ta-Nme1      PVACMVWEG- ---KDVVKTG RRMLGETDPL KS---LPGSI RGDYAIDLG- RNVCHGSDSV ESANKEIKLW FNEDE
Ta-Nme5      PIVAYILAK- ---NNAIEDW RNSMGPTNSM NARIAAPESL RAKYGIDEM- RNGFHGSDGP LTAEREIRFF F----
Ta-Nme6      PATIAILVG- ---NNAITHW RDLIGPSRSH RARSSHPSTI RAIYGLTDT- RNAVHGSDSV ESAAREIQFF F-PE-
Ta-Nme7A     PVLGMELMR- ---SNAIKRW RELLGPTNSS KARQEAPNSI RARYGTDGT- QNACHGSDST DSAAREIEFI F-PT-
Ta-Nme8B     PLVALALAK- ---QDSVDAW RDMIGPPDVN LAKELAPSSL RARYSSDD-- VNVVHGSENH ESAEKELEFF F-PER
Nv-Nme2-a    PVVAMVWEG- ---AGVVKTG RVMLGETNPA DS---KPGTI RGDFCVHIG- RNIIHGSDST DSANKEIALW FSPKE
Nv-Nme2-b    PVCAMVWEG- ---LGVVKTA RVMLGETDPA KS---LPGTI RGDFSIHIG- RNIIHGSDAV ETAKEEIALW FKDDE
Nv-Nme5      PIMALVLAR- ---ENAISYW RQLIGPTNTQ KARDQAPESL RAIYGTDST- RNALHGSDGT VSADKEIHFF F-PDS
Nv-Nme6      PMTAMILGR- ---ENAITHW RKLMGPTHAY KARSIAPKSI RALYGISDT- RNATHGSDSD ESARKEIEFF F-PEF
Nv-Nme7A     PVVAFELKG- ---PGAVDSW RKVLGPTDSA TARNQAPLSV RAKFGTDNT- KNAAHGSDST ESAEREVSFF F-DKR
Nv-Nme8B     PMMALCLAR- ---EDAIEGW RGMLGPKEVE KAKDEAPESL RAQFQVEDSP INPLHGSDTA ENAEKEIQKF F-PM-
```
