# Supplementary material for: New Insights into Placozoan Sexual Reproduction and Development
Source: PLoS One. 2011 May 19;6(5):e19639. doi: 10.1371/journal.pone.0019639 (PMC3098260; doi:10.1371/journal.pone.0019639)
Supplement: Text S1 — Supporting material and methods. (DOC) [file pone.0019639.s005.doc]

**Supporting Material and Methods**

*Animal culturing*

To set up placozoan cultures 50 animals were placed in 2L-aquaria with 3.5% artificial seawater (ASW) at 23°C with a daylight period of 12h under two 30W Osram neon lamp 40cm above the culture. A few milliliters of food from a pure culture of *Pyrenomonas helgolandii* algae (Cryptophycae, Chromalveolata) were added to start cultures. The algae divided autonomously in the culture after addition of soil extract (www.epsag-uni-goettingen.de), KNO_3_ (0.2g/L), K_2_HPO_4_ (20mg/L) and Mg_2_SO_4_ (20mg/L). Under these conditions, placozoans divided continuously until reaching a high density with approximately ten animals per square cm. Starvation and high population density led to a degradation phase (D-phase), to oocyte maturation within 5-6 weeks and finally to growing embryos.

*Generation of Placozoa sp. H4 EST data*

Roughly 2000 healthy growing vegetative animals were used for construction of the cDNA library. Animals were washed three times with sterile 3.5% ASW and starved overnight to prevent algae contamination. Animals were transferred to 1,5ml Eppendorf tubes with approximately 200 animals per tube and ASW was removed after brief centrifugation. Animals were lysed in fresh 500µl homogenisation buffer (HOM: 50mM Tris HCl, 10mM EDTA, 100mM NaCl, 2,5mM DTT, 0,5% SDS, 0.1% DEPC in Ultra pure water (Gibco) at pH 8.0; [S1]). Proteins were digested with 25µg DEPC-treated Proteinase K for 30 minutes at 65°C. The homogenate was forced through a needle connected to a 2.5ml syringe. This step significantly increased nucleic acid yield. Subsequently nucleic acids were isolated by two rounds of Phenol/Cloroform/Isoamylalcohol (25:24:1) purification. Finally DNA was digested with DNaseI (Fermentas) and total RNA was used for cDNA library construction at the MPI for Molecular Genetics (Berlin) using the SMART cDNA Library Construction Kit (Clontech). Initially 4,015 ESTs were 5’ end sequenced, quality and vector clipped and assembled using CAP3 [S2] resulting in 2,196 unique clusters.

*Identification of sperm-associated proteins in placozoans*

To search for genetic spermatogenesis markers in ESTs we used a Blast-based screening. Initially, we screened for obvious markers by searching for the phrases ‘sperm’, ‘testis’ and ‘meiosis’ in the first 10 blast-hits (blastx) of all EST clusters against GenBank protein entries at NCBI (http://blast.ncbi.nlm.nih.gov/Blast.cgi) using default parameters. The resulting list of male gamete-related candidate proteins were blasted against mouse RefSeq proteins at NCBI and filtered for first hits only. This step resulted in two Placozoa sp. H4 orthologs of mouse sperm-associated proteins. Secondly, more mouse sperm-specific proteins were retrieved from GenBank (RefSeq database) and blasted against our EST clusters (tblastn on local Blast server). This led to the identification of three additional homologs of genes related to spermatogenesis in mammals. Homologs to the final five Placozoa sp. H4 candidate sperm-associated proteins in *Trichoplax* *adhaerens* were identified by screening the 27,069 publicly available ESTs (<http://genome.jgi-psf.org/Triad1/Triad1.home.html>, <http://www.ncbi.nlm.nih.gov/nucest>) as well as the 11,514 predicted proteins (JGI).

**Supporting References**

S1. Ender A, Schierwater B (2003) Placozoa are not derived cnidarians: evidence from molecular morphology. *Mol Biol Evol* 20(1):130-134.

S2. Huang X, Madan A (1999) CAP3: A DNA sequence assembly program. Genome Res 9: 868-877.
